# Diffractive small angle X-ray scattering imaging for anisotropic structures

**DOI:** 10.1038/s41467-019-12635-2

**Published:** 2019-11-12

**Authors:** Matias Kagias, Zhentian Wang, Mie Elholm Birkbak, Erik Lauridsen, Matteo Abis, Goran Lovric, Konstantins Jefimovs, Marco Stampanoni

**Affiliations:** 1 Swiss Light Source, Paul Scherrer Institute, 5232 Villigen, Switzerland; 2 Institute for Biomedical Engineering, University and ETH Zurich, 8092 Zurich, Switzerland; 3 Xnovo Technology ApS, 4600 Køge, Denmark; 4 Centre d’Imagerie BioMédicale, École Polytechnique Fédérale de Lausanne, 1015 Lausanne, Switzerland

**Keywords:** Composites, Imaging techniques, X-rays, Imaging and sensing, Micro-optics

## Abstract

Insights into the micro- and nano-architecture of materials is crucial for understanding and predicting their macroscopic behaviour. In particular, for emerging applications such as meta-materials, the micrometer scale becomes highly relevant. The micro-architecture of such materials can be tailored to exhibit specific mechanical, optical or electromagnetic behaviours. Consequently, quality control at micrometer scale must be guaranteed over extended areas. Mesoscale investigations over millimetre sized areas can be performed by scanning small angle X-ray scattering methods (SAXS). However, due to their long measurement times, real time or operando investigations are hindered. Here we present a method based on X-ray diffractive optics that enables the acquisition of SAXS signals in a single shot (few milliseconds) over extended areas. This method is applicable to a wide range of X-ray sources with varying levels of spatial coherence and monochromaticity, as demonstrated from the experimental results. This enables a scalable solution of spatially resolved SAXS.

## Abstract (web summary)

Mesoscale investigations of material microarchitecture using small angle X-ray scattering (SAXS) methods have been limited by long measurement times. Here, the authors present an X-ray diffractive optics method which enables single shot acquisition of SAXS signals over large areas.

## Introduction

The macroscopic mechanical behaviour of materials is directly linked to the arrangement of the individual building elements at a micro or nanoscopic level. Examples of such materials occurring in nature include bone and wood. In both cases the arrangement of fibres provide the mechanical stability or compliance of the structure^1,2^. Bioinspired artificial structures^3–5^ with superior properties such as ultra low weight to strength ratio can be utilised in a wide range of fields such as biomedical engineering^6,7^, aerospace engineering^8^ and the automotive industry^9^. Such structures can be realised with technologies like additive manufacturing and injection moulding where fibrous structures are either mimicked or directly introduced in the raw material. The further development and optimisation of such structures requires accurate understanding of the local fibre orientation which eventually determines the macroscopic mechanical properties. Current technologies capable of probing the fibre orientation at a microscopic level include visible light microscopy, high resolution X-ray computed tomography^10^ and scanning small angle X-ray scattering (SAXS)^11^.

In particular the X-ray based approaches have the benefit to penetrate matter and therefore can image inner or concealed areas. However, both high resolution X-ray computed tomography and spatially resolved SAXS are limited to sample sizes of a few millimetres. In addition, the scan time even with highly brilliant synchrotron beams is in the range of tens of hours^12^, therefore hindering in situ and eventually forbidding real-time or operando investigations. SAXS signals can be partially recorded in the real space over larger areas (centimetre range) by utilising interferometric methods such as X-ray grating interferometry (XGI)^13^. XGI utilises periodic phase modulating structures combined with coherent illumination in order to create an interference fringe at specific distances downstream (Talbot effect). Scattering introduced by the sample causes broadening of the incoming beam and consecutively reduction of the contrast of the interference fringe. The relationship between the reduction in visibility and the small angle scattering properties of the sample has been well developed and understood^14–17^. Conventionally, the interference pattern is limited to only one direction, meaning that only a radial component of the scattering intensity is sensed. For isotropically scattering samples this is not a limitation, however, for the investigation of samples with anisotropic scattering properties rotation of either the sample or the imaging modality is necessary^18,19^, leading to prolonged acquisition times and thus inhibiting the observation of time evolving effects. Recent developments in XGI have allowed an omnidirectional sensitivity of the scattering signal over the imaging plane in a single shot^20^. The method relies on the direct recording of the interference fringe of an array of circular gratings, each creating a self-image at a specific distance downstream the grating which is scaling with the square of the grating period. In order to observe this effect, sufficient coherence, approximately equal to the grating period at the grating, and high enough resolution, at least half of the interference fringe period, are required. Taking into account the low refraction angles of X-rays we can conclude that the existing method is only applicable on highly coherent sources, such as synchrotrons, in combination with micrometre resolution detectors. Therefore, the applicability of the method is constrained to at best millimetre sized samples that can only be investigated at large scale facilities.

Here we propose a general methodology that provides omnidirectional scattering sensitivity, does not stringently require coherent or monochromatic illumination, and finally does not rely on high resolution detectors. The key advancement is the introduction of an optical element capable of creating an illumination field, by incoherent superposition, that locally encodes the scattering properties with omnidirectional sensitivity. We demonstrate the compatibility of the method with synchrotron sources and conventional micro- and macrofocal X-ray tubes. Enabling real-time investigations and examinations of larger objects. Finally, the method is applied to the investigation of the fibre orientations in reinforced polymers manufactured by injection moulding.

## Results

### Demonstration of working principle

**Fig. 1:**
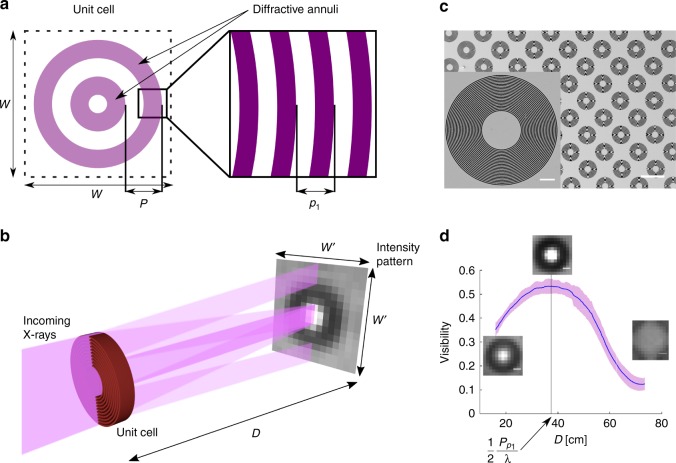
Fringe formation for a single unit cell. **a** Schematic of a single unit cell containing two diffractive annuli. **b** The periodic substructure of the annulus causes diffraction of the incoming X-rays. Given sufficient propagation the $\pm$1 diffraction orders have split and superimpose incoherently with the opposite orders generating the shown intensity modulation pattern. **c** Fabricated grating for $17\ {\rm{keV}}$ containing one diffractive annulus of $50\ \upmu {\rm{m}}$ period and $1\ \upmu {\rm{m}}$ diffractive substructure, the scale bars for the inset and the overview image are 10 and $100\ \upmu {\rm{m}}$, respectively. **d** Measured visibility for the grating in **c** in dependency on the propagation distance $D$. The maximum visibility is observed at a distance of $\frac{1}{2}\frac{P p_{1}}{\lambda }$ independently of the type of the diffractive structure. The insets represented the fringe from a single unit cell at the given distances. The scale bars correspond to $20\ \upmu {\rm{m}}$. The width of the band corresponds to six times the observed standard deviation over the full field of view

The optical element is composed of unit cells that cover the full field of view (FoV) to be examined. Each unit cell has a width of $W$ and is an ensemble of equally spaced concentric annuli with a period $P$, with each annulus containing a periodic diffractive substructure with a period ${p}_{1}$ significantly smaller than the width of the annuli. A schematic representation of a unit cell with two diffractive annuli is shown in Fig. 1a. The width of the annulus can be chosen freely, however, for simplicity we assume that it is half of the coarse period $P$. The diffraction from the substructure results in a conical beam splitting. Provided sufficient propagation distance after the optical element, a modulation pattern with a circular symmetry will start to appear as illustrated in Fig. 1b for the case of a single unit cell with only one diffractive annulus. For clarity, only a cross section of the $\pm 1$ diffraction orders is shown while neglecting the zeroth order. The projected unit cell size $W^{\prime}$ defines the pixel size in the retrieved images and is refereed to as a macropixel. For a period of ${p}_{1}$ of the substructure, the diffraction angle of the $\pm 1$ orders for perpendicularly incident radiation of wavelength $\lambda$ is $\pm {\sin }^{-1}\left(\frac{\lambda }{{p}_{1}}\right)$. In the hard X-ray regime where $\lambda \sim 1{0}^{-11}\,\,\text{m}\,$ and for micrometre sized structures ${p}_{1} \sim 1{0}^{-6}\,\,\text{m}\,$ the diffraction angles can be approximated by $\pm \frac{\lambda }{{p}_{1}}$. The distance at which the $\pm 1$ orders have separated completely and overlap with the transmitted X-rays from the non diffractive areas, and subsequently the intensity of the modulation maximises, is given by $D=\frac{1}{2}\frac{P p_{1}}{\lambda }$ for a parallel beam geometry. A theoretical derivation of the conditions for maximising the visibility is presented in the Methods section. If a sample that exhibits small angle scattering is placed before the optical element, the visibility of the recorded pattern will be reduced. In the Methods section, it is analytically derived that the visibility reduction can be extracted under all angles on the imaging plane and corresponds to a ring of the real space correlation of the sample at a length scale $\xi =\frac{z\lambda }{P}$. Therefore, this type of measurement has the capability to extract the orientation of the underlying scattering structures. For full SAXS analysis measurements at additional correlation lengths would be required. In Fig. 1c a scanning electron microscopy (SEM) image of fabricated optics containing a single diffractive annulus with a fine diffractive period of 1 μm, coarse period of 35.75 μm, and design energy of $17\ {\rm{keV}}$ are shown. The measured average visibility illustrated in Fig. 1d at $17\ {\rm{keV}}$ for distances between 16 and 75 cm between the optical element and the detector, shows a clear maximum of 52% at the theoretical position. The insets depict the intensity pattern of a single unit cell at the marked distances.

### Imaging of injection moulded fibre reinforced polymers

**Fig. 2:**
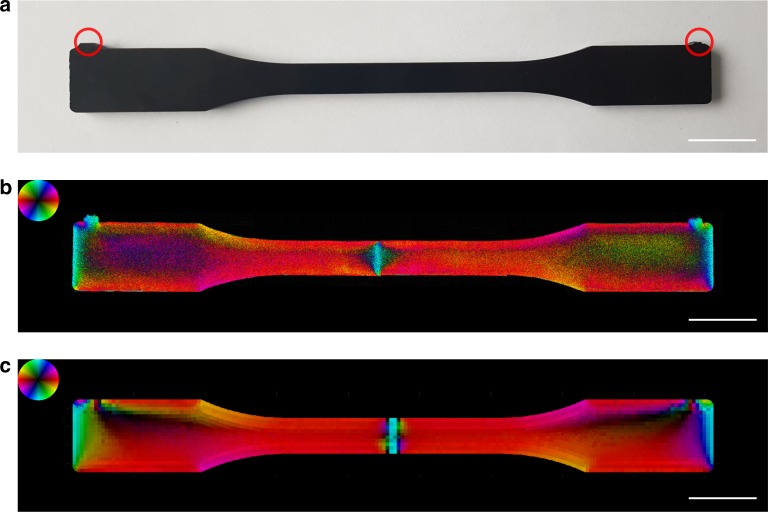
Imaging of fibre orientation in reinforced polymers. The glass fibre reinforced tensile sample (**a**) was injection moulded from the two inlets marked by the red circles creating a weld line near its centre. The extracted fibre orientation map (**b**) was compared with the finite element flow simulation shown in (**c**). In general a high agreement between the simulation and the measurement is observed. However, the simulation fails to predict the exact location and shape of the weld line which is a highly critical area. The scale bars correspond to $2\ {\rm{cm}}$

To demonstrate the capability of the method to extract the fibre orientation in relevant samples we imaged a glass fibre reinforced injection moulded tensile sample shown in Fig. 2a. The injection procedure took place from the two inlets annotated with red circles, thus creating a weld line near the centre of the sample. In Fig. 2b the retrieved orientation and degree of anisotropy of the fibres are shown. The colour corresponds to the orientation according to the colour wheel, and the degree of anisotropy to the intensity. The experimental details and retrieval method are given in the Methods section. In Fig. 2c the corresponding orientation map calculated from a finite element flow simulation is shown. The details of the simulation are given in the Methods. In general, there is a strong agreement between the orientation predicted from the proposed method and the state of the art simulation method. However, the most prominent and crucial difference can be found at the weld line. The simulation fails to predict the accurate location of the weld line, potentially due to the assumption that the injection is happening with exactly the same pressure from both inlets. In addition, our measurements show an asymmetry of the weld line in the horizontal axis, which the simulation failed to predict.

**Fig. 3:**
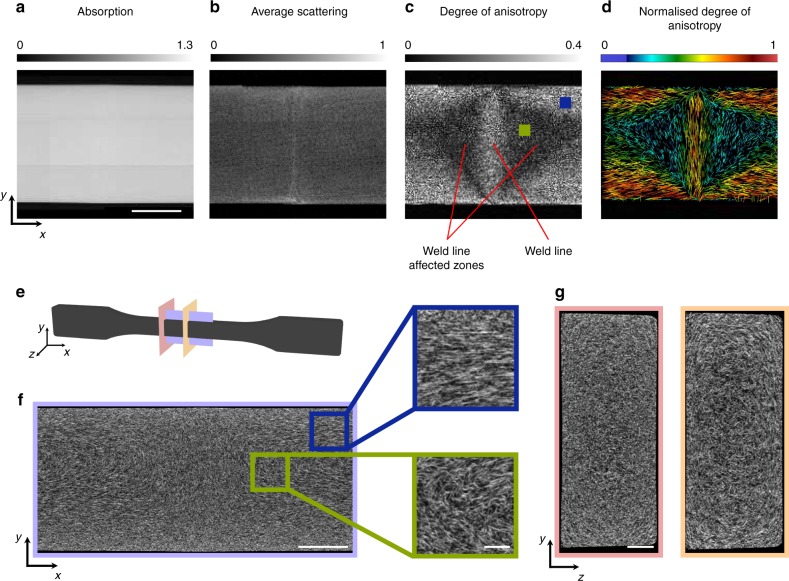
Correlative investigation of weld line. **a** Absorption, **b** average scattering, **c** degree of anisotropy, and **d** retrieved projected fibre orientation images of the weld line (scale bar: 5 mm). In order to validate the observed results, a high resolution tomographic scan was performed around the centre of the tensile sample where the weld line is located. The location of the investigated tomographic slices are shown in (**e**). In the coronal slice (**f**) the asymmetry of the weld line which is observed in the degree of anisotropy image (**c**) can be justified (scale bar: 2.7 mm). The zoom-ins (scale bar: $400\ \upmu {\rm{m}}$) highlight areas of low and high fibre alignment which correlate well with similar areas in the degree of anisotropy image marked by blue and green squares, respectively. The sagittal slices (**g**) show the difference between the weld line affected and non-areas of the sample as the fibre orientation changes from out of plane to in plane (scale bar: 1  ${\rm{mm}}$)

We further investigated the weld line area by comparing the additional contrasts that are available through the proposed method. In the conventional absorption image in Fig. 3a no visible sign of the weld line or any micro-structural difference can be inferred since a uniform absorption signal is observed. On the other hand, looking at the average scattering image shown in Fig. 3b, it becomes evident that some non uniformity is present. Finally, clear evidence is provided by extracting the degree of anisotropy image shown in Fig. 3c. The weld line becomes highly visible together with the neighbouring affected zones, where a significantly lower fibre alignment is inferred. In Fig. 3d the local fibre orientation and degree of anisotropy is plotted with a vector plot, the direction corresponds to the estimated fibre direction and the colour to the degree of anisotropy.

In order to further support the observed difference in the weld line we performed a micrometre computed tomography ($\mu {\rm{CT}}$) scan of the affected area. The Experimental details of the $\mu {\rm{CT}}$ are given in the Methods section. We chose to show three slices, one in the $xy$ plane going through the middle of the sample at the location of the weld line, and two in the $yz$ plane, one directly at the weld line and one $1\ {\rm{cm}}$ further down in the unaffected area. In Fig. 3e the illustrated tomographic slices are marked on a sketch of the tensile sample. The sagittal slice from the unaffected area shows the fibres to be oriented along the $x$ direction (perpendicular to the observation plane). But as we move the observation plane closer to the weld line an increasing number of in plane fibres start to show up. Finally, once the centre of the weld line has been reached all fibres seem to be located in plane as shown in the second sagittal plane in Fig. 3g. From the coronal slice shown in Fig. 3f a deeper understanding of the observed degree of anisotropy shown in Fig. 3c can be accomplished. The zoom-ins correspond to areas of high and low degree or anisotropy marked by the blue and green boxes, respectively. As expected the blue box shows fibres highly aligned while the green fibres more randomly distributed. The observed asymmetry of the weld line can also be justified from the coronal slice, as a higher amount of aligned fibres can be observed at the bottom of the weld line. Finally, the exact apparent shape of the weld line might not match completely the one extracted from the slice due to the fact that we are looking at a projection and not a tomographic reconstruction of the fibre orientation.

### Demonstration of real time fibre orientation extraction

**Fig. 4:**
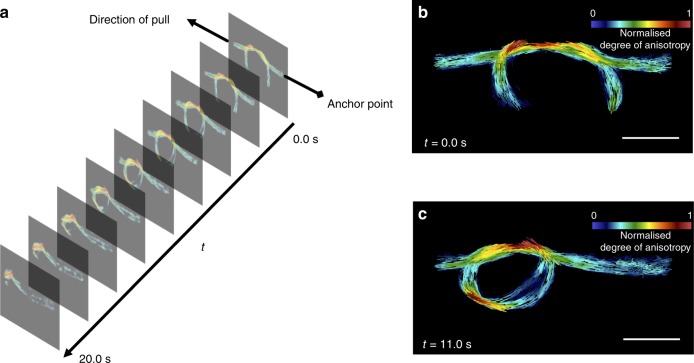
Real-time omnidirectional scattering imaging. **a** Schematic of the experimental setup with an ensemble of images encoding the underling fibre orientation and the degree of anisotropy. The fibre knot was pulled from one end while being anchored from the other. The images were collected with a frame rate of 25 frames per second for a total duration of 20 s. Extracted images at two different time points (**b**) $t = 0.0$ s and (**c**) *t* = 11.0 s. The scale bars correspond to $4\ {\rm{mm}}$

The combination of transparent optics, single shot acquisition mode, and high flux beams from synchrotron sources allow the imaging procedure to be executed in a few milliseconds, thus enabling real-time imaging. As a demonstrator we chose to image a carbon fibre overhand knot in real time (25 frames per second) while it was being tightened. The tensile experiment is sketched in Fig. 4a. The one end of the knot was pulled while the other was anchored to a fixed point. In Fig. 4b and c, the retrieved orientation maps of the carbon fibre knot at time points 0.0 and 11.0 s are shown. The lines correspond to the retrieved direction of the carbon fibres and the colour to the normalised degree of anisotropy (by 0.27) translating in this case directly to the local fibre density variations^18^. In the Supplementary Movie 1 the orientation of the underlying fibres and the local fibre density of the knot throughout the whole experiment are shown. Since only one end of the knot is being pulled a sliding motion in the direction of pull is observed. This causes the free part of the knot to develop an asymmetric pattern, with the left side containing fibres more densely packed than the right. This is observed by the increase in the degree of anisotropy. The intertwined part of the knot shows a stronger signal due to the overlapping fibres of the strands.

### Imaging on conventional X-ray tubes

**Fig. 5:**
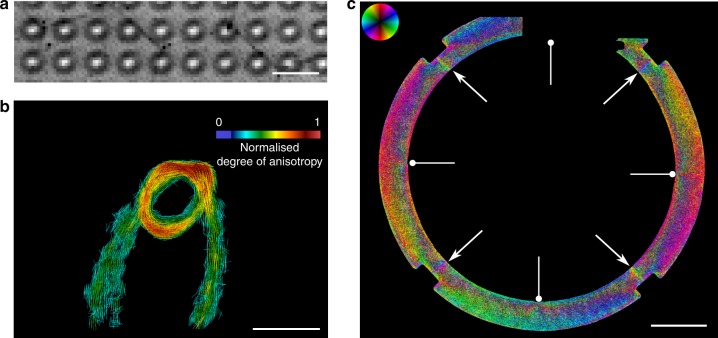
Scattering imaging on polychromatic X-ray source. **a** Recorded intensity modulation on polychromatic X-ray source operated at $70\ {\rm{kVp}}$ with an average visibility of 20%. The scale bar corresponds to $260\ \upmu {\rm{m}}$. **b** Proof of principal image of a carbon fibre loop. The rendered lines represent the reconstructed direction of the underlying fibres while the colour encodes the degree of anisotropy. The scale bar is $4\ {\rm{mm}}$. **c** Reconstructed orientation map of an injection moulded carbon fibre reinforced component. The pointy arrows correspond to the occurring weld lines while the rounded head arrows to the injection points. The scale bar is equal to $2\ {\rm{cm}}$

For the translation of the method from synchrotron facilities to laboratory X-ray sources two major challenges need to be considered: source size and the polychromatic spectrum of such sources. Intrinsically, the method does not rely on coherent illumination, however, the projected source size should be small enough in order not to completely blur out the generated pattern. Practically, this can be avoided if the projected source size is kept smaller than half of the projected pattern’s period $P^{\prime}$, leading to the condition:

1 $$\sigma \, < \, \frac{1}{2}\frac{L}{D}P,$$

where *L* is the source to detector distance and $\sigma$ the width of the X-ray source. Given that condition (1) is not usually fulfilled, an aperture array can be introduced in front of the source to mitigate this effect. The opening of the apertures should be small enough in order to fulfil condition (1), and their spacing such that a superimposition of the individual generated patterns corresponding to each aperture is achieved (Lau condition)^21^. In Supplementary Note 1, we present proof of concept images recorded with a macrofocal X-ray tube ($1\ {\rm{mm}}$) in combination with an aperture array. In addition, discussion regarding the flux efficiency of such an approach is presented. Regarding the polychromaticity it is demonstrated in the Methods section that theoretically a spectral acceptance of 90% can be achieved, therefore confirming the achromatic character of the method and its compatibility with a wide range of X-ray sources. In Fig. 5a, a recorded intensity modulation with a visibility of 20% is shown resulting from a microfocal tube operated at $70\ {\rm{kVp}}$. As a demonstrator we imaged a carbon fibre loop with predictable fibre orientation. As shown in Fig. 5b, a reliable reconstruction of the fibre orientation was achieved. Fig. 5c presents the fibre orientation map of a carbon fibre reinforced injection moulded component and well illustrates the application of our method to an industrially relevant sample. Four injection locations were used, indicated by the round head arrows. The top part of the sample was not imaged due to geometric constrains of our setup. The pointy arrows mark the resulting weld lines.

## Discussion

In this work we introduced a generalised imaging method for obtaining scattering signals with an omnidirectional sensitivity at a defined correlation length. Due to the lack of any strong coherence or monochromaticity requirements the method is compatible with a wide range of X-ray sources and X-ray detectors as demonstrated in the experimental section.

The presented experiments demonstrate the potential of the method to address current challenges in the field of fibre reinforced structures where both fibre orientation and density play a crucial role in the observed macroscopic mechanical properties. It could do so by being integrated in the development, quality control, and or testing cycles. Thus overcoming the limitation of the current state of the art simulative methodology. Nevertheless, the applicability of the method is not only limited to fibre reinforced polymer structures, but can be extended to biological specimens such as bone^22^ or brain tissue^23^. On a more speculative basis, the compatibility with high power and energy X-ray tubes opens up the possibility to image hard condensed systems such as alloys. Potentially, lamellar orientation could be extracted which again is crucial for the mechanical properties^24^ of the alloys. On the soft matter side, applications could include imaging of three dimensional printed structures^25^ such as biologically inspired composites, shape-morphing systems, and soft sensors.

The length scale sensitivity of our method is equal to half of the period of the diffractive structure; with the current fabrication methods of X-ray optics periods spanning between a few micrometres down to hundreds of nanometres^26^ can be implemented. In addition, the method optimally (in terms of fringe visibility and photon utilisation) requires phase shifting gratings, by utilising heavier materials such as Ir or Au optics for high energies can be fabricated and thus targeting either samples of industrial scale or hard condensed matter.

The degree of coherence of the X-ray sources plays an integral role in determining the final fringe visibility since geometric blurring needs to be taken into account. Therefore, highly coherent sources will allow for an increased flat visibility. The noise level of the retrieved scattering signal is directly linked to the nominal visibility^27^. Thus, we foresee that an increase in coherence, which is expected with the upcoming machine upgrades of light sources, can benefit the method in two ways: either by allowing to reduce the acquisition time in order to observer faster dynamic effects, or allow for an increase in sensitivity for weakly scattering materials such as biological samples.

The main limitation of the current method is the fixed autocorrelation length. Meaning that in oder to ripe the benefits of full SAXS quantification methodologies^28^ additional measurements at different autocorrelation lengths are required. In order to avoid this, a different unit cell design could be implemented allowing multiple autocorrelation lengths in a single shot (M. Kagias, Z. Wang, G. Lovric, K. Jefimovs and M. Stampanoni, manuscript in preparation).

The single shot aspect of the method unlocks the potential for either time resolved studies with millisecond temporal resolution over centimetre sized areas, previously impossible with conventional SAXS methods, or accelerated tensor tomographic imaging with acquisition times in the range of a few seconds compared with hours with the current methods. Finally, the method can also be extended to other forms of radiation from low coherence and brilliance sources such as neutrons.

## Methods

### Visibility calculation and design optimisation

We perform a theoretical analysis of the visibility estimation assuming that the diffractive substructure is composed of a binary grating with complex transmissions of ${T}_{h}$ and ${T}_{l}$, duty cycle ${d}_{c}=\frac{{p}_{h}}{{p}_{h} \, + \, {p}_{l}}$, with ${p}_{h}$ and ${p}_{l}$ being the lengths of the two states, respectively, and ${p}_{1}={p}_{h}+{p}_{l}$. Our goal is to extract optimum parameters for maximising the fringe visibility. The diffraction efficiencies can be calculated from the coefficients of a Fourier series expansion of the diffractive substructure. The efficiencies for the $| n| \ge 1$ orders are given by

2 $${A}_{n}=\frac{1}{2}{\left(\frac{1}{\pi n}\right)}^{2}| {T}_{h}-{T}_{l}{| }^{2}[1-\cos (2\pi n{d}_{c})]$$

The transmitted order is given from

3 $${A}_{0}=| {T}_{h}{d}_{c}+(1-{d}_{c}){T}_{l}{| }^{2}$$

For simplicity we will consider only a radial cross section of a single unit cell of the optical element. After a propagation distance $z$ the recorded intensity $I(x)$ can be considered as the sum of the non diffracted ${I}_{0}(x)$ and diffracted ${I}_{n}(x)$ orders

4 $$I(x)={I}_{0}(x)+\sum _{n= \pm 1,\pm 2,...}^{\pm \infty }{I}_{n}(x)$$

The visibility is estimated as twice the ratio of the first and zeroth Fourier components of the recorded intensity. Due to the linearity of Fourier series, we can calculate the zeroth and first Fourier components for each diffraction order separately, then perform the addition in the Fourier space. Each intensity ${I}_{n}(x)$ can be considered as a pulse train. The zeroth order pulse train will have levels of 1 and ${A}_{0}$, in the case of a generic order $n$, the pulse train will have levels of $0$ and ${A}_{n}$ incorporating a shift of ${s}_{n}=n\frac{\lambda }{{p}_{1}}z$. Taking into account the above assumption we conclude that the zeroth order Fourier component will be given by

5 $${\alpha }_{0}=\frac{1+{A}_{0}+2{\sum }_{n=1,2,...}^{\infty }{A}_{n}}{2}$$

And the first by

6 $${\alpha }_{1}=\frac{2}{\pi }[1-{A}_{0}-2\sum _{n=1,2,...}^{\infty }{A}_{n}\cos (2\pi {s}_{n}\frac{n}{P})]$$

Resulting in a visibility of

7 $$V(z)=\frac{4}{\pi }\frac{1-{A}_{0}-2{\sum }_{n=1,2,...}^{\infty }{A}_{n}\cos (2\pi {n}^{2}\frac{\lambda }{P{p}_{1}}z)}{1+{A}_{0}+2{\sum }_{n=1,2,...}^{\infty }{A}_{n}}$$

Our goal is to estimate the distance $z$ at which the visibility will maximise. The positions where the visibility maximises or minimises are given from the solutions of the following equation

8 $$\frac{\partial V(z)}{\partial z}=0,$$

which can be rewritten as

9 $$\sum _{n=1,2,...}^{\infty }[1-\cos (2\pi n{d}_{c})]\sin \left(2\pi {n}^{2}\frac{\lambda }{P{p}_{1}}z\right)=0$$

A set of solutions of Eq. (9) can be given when the sinusoidal term is equal to 0 for every $n$. This can be achieved if

10 $$2\pi \frac{\lambda }{P{p}_{1}}{z}_{l}=l\pi ,l\in {\mathbb{N}}$$

In order to find which of these solutions represent maxima we calculate the second order partial derivative of the visibility which should be positive

11 $$\frac{{\partial }^{2}V(z)}{\partial {z}^{2}}{\left.\right|}_{z={z}_{l}}\; > \; 0,$$

which results in the following set of locations where the visibility maximises in the case of monochromatic radiation

12 $${z}_{m}=m\frac{P{p}_{1}}{2\lambda },m=1,3,...$$

These solutions are general and do not depend on the type (phase, attenuation or mixed) of the diffractive structure used but only on the wavelength and periods. Further optimisation can be performed at these locations to maximise the observed visibility. Specifically, we want to optimise for the duty cycle and transmission levels of the diffractive substructures. For simplicity we will focus on the case where the diffractive structure is only inducing a phase shift of $\Delta \Phi$. In this case the diffraction efficiencies of formulas (2) and (3) become

13 $${A}_{n}=2{\sin }^{2}\left(\frac{\Delta \Phi }{2}\right){\left(\frac{1}{\pi n}\right)}^{2}[1-\cos (2\pi n{d}_{c})],$$

and

14 $${A}_{0}=1-4{d}_{c}(1-{d}_{c}){\sin }^{2}\left(\frac{\Delta \Phi }{2}\right)$$

In addition, the denominator of the second fraction of the visibility term according to Eq. (7) will converge to 2 since no intensity is lost in the grating structure. From the above we can straightforwardly conclude that for pure phase shifting optics the visibility maximises when the phase shift $\Delta \Phi$ is an odd multiple of $\pi$ and the duty cycle is 0.5. In the case of mixed gratings optimum visibility is expected for different duty cycle and phase/transmission values.

### Spectral acceptance

In the previous section we derived the conditions for maximising the visibility in the case of monochromatic radiation. Here we will demonstrate that fringe formation process still takes place with a broad spectrum. For the sake of simplicity we will assume a purely phase shifting element, with a duty cycle of 0.5, with energy dependent refractive index decrement of $\delta (\lambda )$, designed to introduce a phase shift of $\pi$ at a given wavelength ${\lambda }_{d}$ or energy ${E}_{d}$. In addition, we will take into account only the $\pm 1$ diffraction orders. At the nominal design distance of ${z}_{1}=\frac{1}{2}\frac{P{p}_{1}}{{\lambda }_{d}}$ from Eqs. (7), (13), and (14) the visibility becomes

15 $$V(E)=\frac{2}{\pi }{\sin }^{2}\left(\frac{\pi }{2}\frac{E}{{E}_{d}}\frac{\delta (E)}{\delta ({E}_{d})}\right)\left[1-\frac{8}{{\pi }^{2}}\cos \left(\pi \frac{{E}_{d}}{E}\right)\right]$$

Under the assumption that the refractive index decrement is inversely proportional to the square of the photon energy $\delta (E)\propto 1/{E}^{2}$ (which holds true in the absence of absorption edges etc.) Eq. (15) becomes

16 $$V(E)=\frac{2}{\pi }{\sin }^{2}\left(\frac{\pi }{2}\frac{{E}_{d}}{E}\right)\left[1-\frac{8}{{\pi }^{2}}\cos \left(\pi \frac{{E}_{d}}{E}\right)\right]$$

The spectral acceptance $\Delta E/{E}_{d}$ is calculated as the full width half maximum (FWHM) of the lobe occurring around the design energy ${E}_{d}$, which is calculated to be ~$90 \%$ from Eq. (16). Indicating that our method is highly compatible with polychromatic sources. Nonetheless, the spectral acceptance rapidly reduces once higher order design distances are considered. For example, for $m=3$ and $m=5$ the spectral acceptance becomes $\Delta E/{E}_{d}\approx 34 \%$ and $\Delta E/{E}_{d}\approx 17 \%$, respectively.

### Modelling of interaction between sample and optical element

Let us assume that the sample exhibits uniform scattering properties within the area of a single unit cell. This scattering behaviour is described by the symmetric two dimensional scattering function $S({q}_{x},{q}_{y})$ with ${q}_{x}$ and ${q}_{y}$ being the scattering vectors in the $x$ and $y$ directions, respectively. For convenience we will utilise the polar representation of the scattering function $S(q,\theta )$. If the sample is placed right before the optical element and scatters with a vector $q$ under angle $\theta$, the diffraction orders of the radial cross section of the optical element under angle $\psi$ will propagate with an angle given by

17 $${\psi }_{n}=\frac{\lambda q}{2\pi }\cos (\phi -\theta )+n\frac{\lambda }{{p}_{1}},n\in {\mathbb{N}}$$

By following similar calculations as in the previous subsection it can be shown that the visibility at angle $\phi$ will be scaled by a factor

18 $$\cos \left[\xi q\cos (\phi -\theta )\right],$$

where $\xi =\frac{z\lambda }{P}$. To retrieve the total visibility reduction under angle $\phi$ noted by ${V}_{r}(\xi ,\phi )$ we need to integrate over all scattering vectors

19 $${V}_{r}(\xi ,\phi )=\int_{0}^{\infty} \int_{-\pi }^{\pi }S(q,\theta )\cos \left[\xi q\cos (\phi -\theta )\right]q\,{\text{d}}\theta\, {\text{d}}\,q$$

By changing to the cartesian coordinates ${q}_{x}$, ${q}_{y}$, ${\xi }_{x}$ and ${\xi }_{y}$ the above integral can be written as

20 $${V}_{r}({\xi }_{x},{\xi }_{y})=\int_{-\infty }^{\infty} \int_{-\infty }^{\infty }S({q}_{x},{q}_{y})\cos \left({q}_{x}{\xi }_{x}+{q}_{y}{\xi }_{y}\right)\,{\text{d}}{q}_{x}{\text{d}}\,{q}_{y},$$

which implies that the visibility reduction is the Fourier cosine transform of the scattering function. Nonetheless, it is known that the real space correlation function $G({\xi }_{x},{\xi }_{y})$ and the scattering function form a pair of the Fourier cosine transform

21 $$S({q}_{x},{q}_{y})\mathop{\leftrightarrow }\limits^{{{\mathscr{F}}}_{c}}G({\xi }_{x},{\xi }_{y}),$$

therefore it can be deducted that the visibility reduction corresponds to the real space correlation of the sample. The distance $z$ at which the detector is placed defines the length scales that are being probed. In the case the measurement is carried out at the optimum distance described in the previous subsection, we obtain a ring from the real space correlation function at a correlation length equal to half of the period of the fine diffractive structure.

### Retrieval of the directional visibility reduction

The recorded pattern for each unit cell in the absence of a sample can be approximated as a radial cosine

22 $${I}_{f}(r,\phi )={A}_{f}\left[1+{V}_{f}(\xi ,\phi )\cos \left(2\pi \frac{r}{P}\right)\right],$$

where ${A}_{f}$ corresponds to the average intensity of the flat pattern and ${V}_{f}(\xi ,\phi )$ the radial flat visibility. Under the assumption that the investigated sample has uniform scattering properties in the range of one unitcell, the recorded intensity at each unit cell once the sample has been introduced will be

23 $${I}_{s}(r,\phi )={A}_{s}\left[1+{V}_{s}(\xi ,\phi )\cos \left(2\pi \frac{r}{P}\right)\right]$$

For each recorded unit cell a two dimensional discrete Fourier transform is performed. The transmission of the sample for each unit cell is calculated as the ratio of the zeroth orders of the flat and sample measurements ${T}_{m,n}={\hat{I}}_{s}({{\boldsymbol{q}}}_{m,n}={\boldsymbol{0}})/{\hat{I}}_{f}({{\boldsymbol{q}}}_{m,n}={\boldsymbol{0}})$. The visibility reduction at each angle $\phi$ is then given by

24 $${V}_{r}(\xi ,\phi )=\frac{{V}_{s}(\xi ,\phi )}{{V}_{f}(\xi ,\phi )}=\frac{{\hat{I}}_{s}({{\boldsymbol{q}}}_{m,n}={{\boldsymbol{q}}}_{\phi }^{N}){\hat{I}}_{f}({{\boldsymbol{q}}}_{m,n}={\boldsymbol{0}})}{{\hat{I}}_{s}({{\boldsymbol{q}}}_{m,n}={\boldsymbol{0}}){\hat{I}}_{f}({{\boldsymbol{q}}}_{m,n}={{\boldsymbol{q}}}_{\phi }^{N})},$$

where $N$ is the ratio of the unit cell size $W$ and the coarse period $P$. The angularly dependent visibility reduction can be modelled as following:

25 $${V}_{r}(\xi ,\phi )={\alpha }_{0}+{\alpha }_{1}\cos (\theta -{\phi }_{d}),$$

where ${\alpha }_{0}$ corresponds to the average scattering intensity, ${\alpha }_{1}$ the oriented component of the scattering, and ${\phi }_{d}$ the direction of the underlying structure. The ratio ${\alpha }_{1}/{\alpha }_{0}$ is the degree of anisotropy. These three parameters can be extracted by Fourier analysis.

### Fabrication of the optical elements

The pattern was written with an electron beam tool (Vistec) on $250\ \upmu {\rm{m}}$ thick Si wafers covered with 50 nm of Cr and 250 nm poly(methyl methacrylate) (PMMA). After development the pattern was transferred to the Cr mask by reactive ion etching of Cl and ${\text{CO}}_{2}$ plasm. Finally, the Si was etched to the specified depth depending on the design photon energy.

### Omnidirectional imaging on synchrotron X-ray sources

In both cases monochromatic X-rays, selected by a multilayer monochromator (2% bandwidth) were used. After passing through the sample and the optical element the X-rays were converted to visible light by a 300-μm thick scintillator and finally recorded with a pco.edge 5.5 CMOS camera equipped a pixel size of 6.5 μm. For the tensile sample, the energy was set to $25\ {\rm{keV}}$. The optical element had a unit cell size of 71.5 μm, fine period of 1.2 μm, and a coarse period of 35.75 μm. Each unit cell contained only one annulus. Due to the parallel beam geometry the macropixel size of the retrieved images correspond to the unit cell size of 71.5 μm. The field of view was $1.5\times 0.4\ {{\rm{cm}}}^{2}$ and was limited by the beam size. For this reason multiple exposures were performed in order to image the full sample. For the time resolved experiment, optics designed for $17\ {\rm{keV}}$, with a unit cell size of 84.5 μm, fine and coarse periods of 1 and 42.25 μm, respectively. Again due to the parallel beam geometry a final macropixel size of 84.5 μm was achieved in the retrieved images. The exposure time for each frame was set to 40 ms giving a frame rate of 25 frames per second which is considered real time.

### Omnidirectional imaging on X-ray tubes

The carbon fibre loop was measured with the HAMAMATSU L10101 X-ray tube, operated at $70\ {\rm{kVp}}$ and $100\ \upmu {\rm{A}}$, resulting in a source size of $10\ \upmu {\rm{m}}$. The optical element had a diffractive period ${p}_{1}=1\ \upmu {\rm{m}}$, annular period $P=35.75\ \upmu {\rm{m}}$ and unit cell size $W=71.5\ \upmu {\rm{m}}$. The total system had a length of $1\,\,\text{m}\,$ with the optical element placed in the middle. The sample was placed right before the optical element. Due to the symmetric configuration the macropixel size was also $71.5\ \upmu {\rm{m}}$. The detector in use was the PI-SCX:4300 equipped with a (Gadox) scintillator and a pixel size of $24\ \upmu {\rm{m}}$. The exposure time for the flat and sample images was $60\ \sec$ each. For the carbon fibre reinforced component a similar configuration was used. Instead of a phase shifting optical element we utilised an absorbing structure in order to mitigate beam hardening effects. A mosaic scan was performed to cover the whole structure, a total of 47 images where acquired with at least 50% overlap and exposure time of $10\ \min$ each.

### Simulation of fibre orientation in injection moulded sample

The injection moulding simulation was performed in Moldex3D^29^ and the resulting fibre orientation tensors were exported in a structural finite element mesh for structural simulations in Ansys Workbench. The simulations were performed in a grid with approximately $1\times 1\times 0.5\ {{\rm{mm}}}^{3}$ element sizes. The injection simulation was performed in PA66 GF30 glass-filled polymer with a filling time of 0.6 s, melt temperature of 295 ${}^{\circ }{\rm{C}}$, mould temperature of 100 ${}^{\circ }{\rm{C}}$ and a maximum filling pressure of 500 MPa. The resulting 3D orientation tensor information was projected onto ellipses in the $xy$-plane and averaged over the $z$-direction. The orientation and the degree of orientation for the fibre orientation plot were calculated as the direction of the major-axis and the ratio between major- and minor-axis, respectively.

### Micro CT of weld line

The measurement was done using a ZEISS Xradia 520 Versa X-ray microscope with a Flat Panel (FPX) detector. The polychromatic micro-focus source was operated at 80 kV, 7 W and with a low energy filter (LE2) to optimise transmission. In total, 3201 radiographs were acquired over 360 sample rotation with an exposure time of 1 s per radiograph, resulting in a total scan length of 1 h 30 min. The transmission images were reconstructed using the commercial software from ZEISS to an isotropic voxel size of $12.5\ \upmu {\rm{m}}$.

## Supplementary information

Supplementary Information

Description of Additional Supplementary Files

Supplementary Movie 1

## Data Availability

The data sets generated and analysed during this study are available from the corresponding author upon reasonable request.

## Code availability

The code used for data analysis as well as for display of the data is available from the corresponding author upon reasonable request.

## References

[1] Currey, J. D. *Bones Structure and Mechanics* (Princeton Univ. Press, 2006).

[2] Song J (2018). Processing bulk natural wood into a high-performance structural material. Nature.

[3] Gao H, Ji B, Jager IL, Arzt E, Fratzl P (2003). Materials become insensitive to flaws at nanoscale: lessons from nature. Proc. Natl Acad. Sci. USA.

[4] Meyers MA, McKittrick J, Chen P-Y (2013). Structural biological materials: critical mechanics-materials connections. Science.

[5] Martin JJ, Fiore BE, Erb RM (2015). Designing bioinspired composite reinforcement architectures via 3D magnetic printing. Nat. Commun..

[6] Petersen A (2018). A biomaterial with a channel-like pore architecture induces endochondral healing of bone defects. Nat. Commun..

[7] Lee VK, Dai G (2017). Printing of three-dimensional tissue analogs for regenerative medicine. Ann. Biomed. Eng..

[8] Balakrishnan, P., John, M., Pothen, L., Sreekala, M. & Thomas, S. In *Advanced Composite Materials for Aerospace Engineering*, 365–383 (Elsevier, 2016).

[9] Puglia D, Biagiotti J, Kenny JM (2005). A review on natural fibre-based composites—Part II. J. Nat. Fibers.

[10] Uzun sH, Malkoç MA, Keleş A, Öreten AT (2016). 3D micro-CT analysis of void formations and push-out bonding strength of resin cements used for fiber post cementation. J Adv Prosthodont..

[11] Bunk O (2009). Multimodal x-ray scatter imaging. New J. Phys..

[12] Liebi M (2015). Nanostructure surveys of macroscopic specimens by small-angle scattering tensor tomography. Nature.

[13] Pfeiffer F (2008). Hard-X-ray dark-field imaging using a grating interferometer. Nat. Mater..

[14] Yashiro W, Terui Y, Kawabata K, Momose A (2010). On the origin of visibility contrast in x-ray Talbot interferometry. Opt. Express.

[15] Lynch SK (2011). Interpretation of dark-field contrast and particle-size selectivity in grating interferometers. Appl. Opt..

[16] Strobl M (2015). General solution for quantitative dark-field contrast imaging with grating interferometers. Sci. Rep..

[17] Gkoumas S (2016). A generalized quantitative interpretation of dark-field contrast for highly concentrated microsphere suspensions. Sci. Rep..

[18] Jensen TH (2010). Directional x-ray dark-field imaging of strongly ordered systems. Phys. Rev. B.

[19] Jensen TH (2010). Directional x-ray dark-field imaging. Phys. Med. Biol..

[20] Kagias M, Wang Z, Villanueva-Perez P, Jefimovs K, Stampanoni M (2016). 2D-Omnidirectional hard-X-ray scattering sensitivity in a single shot. Phys. Rev. Lett..

[21] Jahns J, Lohmann A (1979). The Lau effect (a diffraction experiment with incoherent illumination). Opt. Commun..

[22] Jud C (2017). Trabecular bone anisotropy imaging with a compact laser-undulator synchrotron x-ray source. Sci. Rep..

[23] Wieczorek M (2018). Brain connectivity exposed by anisotropic X-ray dark-field tomography. Sci. Rep..

[24] Palomares-García AJ, Pérez-Prado MT, Molina-Aldareguia JM (2017). Effect of lamellar orientation on the strength and operating deformation mechanisms of fully lamellar TiAl alloys determined by micropillar compression. Acta Mater..

[25] Truby RL, Lewis JA (2016). Printing soft matter in three dimensions. Nature.

[26] Miao H, Gomella AA, Chedid N, Chen L, Wen H (2014). Fabrication of 200 nm period hard X-ray PHase Gratings. Nano Lett..

[27] Chabior M (2011). Signal-to-noise ratio in x ray dark-field imaging using a grating interferometer. J. Appl. Phys..

[28] Feigin LA, Svergun DI (1987). Structure Analysis by Small-Angle X-Ray and Neutron Scattering.

[29] Moldex3D. http://www.moldex3d.com/en

