# Supplementary material for: Diffractive small angle X-ray scattering imaging for anisotropic structures
Source: Nat Commun. 2019 Nov 12;10:5130. doi: 10.1038/s41467-019-12635-2 (PMC6851111; doi:10.1038/s41467-019-12635-2)
Supplement: Supplementary file 1 — Supplementary Information [file 41467_2019_12635_MOESM1_ESM.pdf]

Supplementary Information for
"Diffractive small angle X-ray scattering imaging for anisotropic structures"

Matias Kagias[1], Zhentian Wang[1,2], Mie Elholm Birkbak[3], Erik Lauridsen[3], Matteo Abis[1,2], Goran Lovric[1,4], Konstantins Jefimovs[1], and Marco Stampanoni[1,2]

[1] *Swiss Light Source, Paul Scherrer Institute, 5232 Villigen, Switzerland*

[2] *Institute for Biomedical Engineering, University and ETH Zurich, 8092 Zurich, Switzerland*

[3] *Xnovo Technology ApS, 4600 Køge, Denmark*

[4] *Centre d'Imagerie BioMédicale, École Polytechnique Fédérale de Lausanne, 1015 Lausanne, Switzerland*

I. SUPPLEMENTARY NOTE 1: IMAGING ON MACROFOCAL X-RAY SOURCES

The compatibility of the method was demonstrated by imaging a carbon fibre loop on a macrofocal source in combination with a source array shown in 1 (a). In figure 1 (b) we show the recorded intensity pattern exhibiting a visibility of 30%. Finally, the retrieved fibre orientations are shown in figure 1 (c). We utilised the MXR-225/26 X-ray tube operated at 60 kVp and 10 mA resulting in a source size of 1 mm. The pinhole array was placed right in front of the X-ray tube and was fabricated on a 200 µm thick tungsten plate by ps-laser ablation. The pinholes had a diameter ranging from $s = 50\,\mu\text{m}$ to $s = 100\,\mu\text{m}$, with a variance given by the penetration of the laser through the tungsten plate. The period of the pinholes was $W_0 = 455.5\,\mu\text{m}$ in order to satisfy the Lau condition. The optical element is made out of Si and had the following parameters: $p_1 = 2\,\mu\text{m}$, $P = 150\,\mu\text{m}$, $W = 300\,\mu\text{m}$, and etch height 37 µm. The exposure time for both flat and sample images was set to 100 sec. The pinhole array to optical element distance was 57 cm and from the optical element to the X-ray detector 110 cm. The detector used is a hybrid photon counting (HPC) SANTIS prototype provided by DECTRIS Ltd. It has a 0.75 mm thick CdTe sensor, a pixel size of 75 µm and two energy thresholds. By setting the energy threshold above 6 keV the images acquired are electronic and readout noise free. For the purpose of our experiments, the HPC detector was operated in single threshold mode. The threshold was set at 20 keV to limit the contribution of charge sharing and effectively provide a sharp square-like point spread function for each pixel. Assuming a maximum pinhole opening such that the projected width of the pinhole is equal to half of the projected annular period we can calculate an intrinsic flux efficiency of

$$\frac{I}{I_0} = \frac{\pi}{16}\left(\frac{P}{W}\right)^2, \tag{1}$$

where W is the width of the unit cell, I_0 is the flux before the source array and I the flux after the array. The maximum flux efficiency is obtained when minimising the ratio between the unit cell size and the annular period. The minimum achievable value is 2 and therefore the maximum efficiency is equal to $\pi/64 \approx 5\%$. Nonetheless, by imposing less stringent conditions for the width of the apertures higher flux efficiencies can be achieved. Depending on the application in mind a trade off between visibility loss and flux efficiency can be imposed.

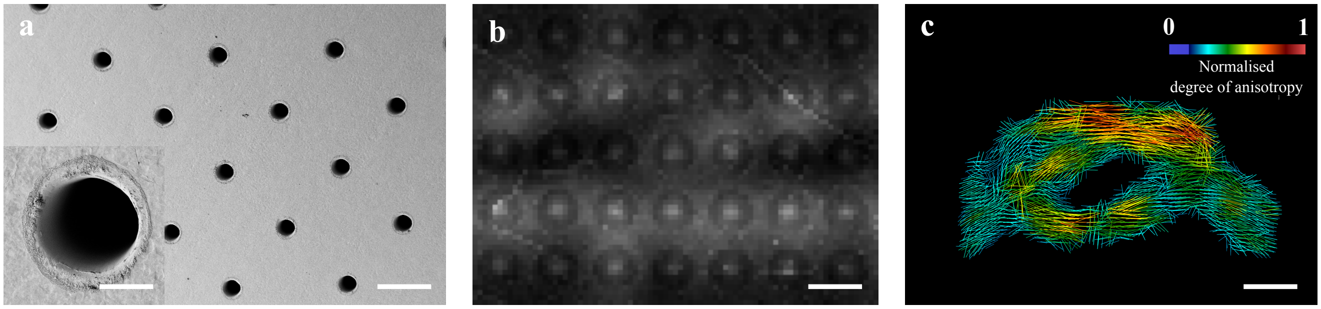

Supplementary Figure 1: **Omnidirectiona imaging on macrofocal X-ray tubes a** Scanning electron image of the fabricated source array. The scale bars correspond to 300 µm for the main image and 20 µm for the insert. **b** Recorded intensity pattern resulting in a visibility of approximately 30%, the scale bar corresponds to 825 µm. **c** Retrieved orientation map of carbon fibre loop sample, the scale bar corresponds to 4 mm.
