# Supplementary material for: Diffractive small angle X-ray scattering imaging for anisotropic structures
Source: Nat Commun. 2019 Nov 12;10:5130. doi: 10.1038/s41467-019-12635-2 (PMC6851111; doi:10.1038/s41467-019-12635-2)
Supplement: Supplementary file 2 — Description of Additional Supplementary Files [file 41467_2019_12635_MOESM2_ESM.pdf]

Description of Additional Supplementary Files

File Name: Supplementary Movie 1
Description: Real time movie of carbon fibre knot while being tightened. The lines correspond to the retrieved direction of the scattering fibres and the colours to the scattering intensity.
